# Frequency and Timing of Specialized Formula Prescriptions for Children Under 2 Years

**DOI:** 10.1111/cea.70447

**Published:** 2026-10-07

**Authors:** Nikita Punjabi, Karen Li, Mark Cunningham, Kimberley Foley, Frank Moriarty, Sonia Saxena, Jennifer K. Quint, Dougal Hargreaves, Robert J. Boyle

**Affiliations:** ^1^ School of Public Health Imperial College London London UK; ^2^ School of Pharmacy and Biomolecular Sciences RCSI University of Medicine and Health Sciences Dublin Ireland

## Abstract

**Background:**

Infants with non‐specific symptoms are sometimes labelled as cow's milk allergic and managed with specialized formula. These products differ from standard infant formula in protein and carbohydrate source, typically containing lower quality protein and free/added sugars such as glucose polymers or sucrose. We evaluated the frequency, timing, duration and estimated volume of specialized formula prescribed to young children.

**Methods:**

We undertook a retrospective population‐based cohort study of children up to age 2 years born between 2009 and 2021 who were registered with a contributing UK general practice, predominantly in England. Community prescribing and demographic data were obtained from the Clinical Practice Research Datalink (CPRD) Aurum, a de‐identified primary care database representing approximately 24% of the population. National prescribing data for England were used to estimate community prescribing rates for 2022–2025.

**Results:**

Of 4,203,276 children born between 2009–2021 in the database, 1,944,323 (46.3%) were registered by age 3 months with complete data to age 2 years and were included. Of these, 111,478 (5.7%) were prescribed specialized formula, rising from 1.7% of children born in 2009 to 7.9% in 2021 and an estimated 9.7% in 2025. Prescriptions were most commonly initiated at age 0–2 months. Multiple prescriptions were documented for 85,370 (76.6%) of these children. Children with multiple amino‐acid formula prescriptions consumed an estimated mean total of 242 L; those with multiple extensively hydrolysed formula prescriptions 203 L; and those with multiple soya formula prescriptions 137 L. Among these children, the median interval between first and last prescription was 257 days for amino‐acid formula, 213 days for extensively hydrolysed formula and 152 days for soya formula.

**Conclusions:**

Community prescribing of specialized formula in England increased approximately five‐fold over the study period, potentially triggering significant free sugars exposure during infancy.

## Key Messages

- Community prescribing of specialized formula increased over 5‐fold from 2009 to 2025 in England.
- Prescriptions were most commonly initiated at age 0–2 months and usually continued for 5–8 months.
- These data suggest that exposed infants consume large quantities of free sugars via specialized formula.

## Graphical abstract

We analysed routinely collected primary care data from the United Kingdom for children aged under 2 years. We found prescriptions for specialized formulas such as extensively hydrolysed formula or amino‐acid formula increased five‐fold between 2009 and 2025. This represents an important increase in free/added sugars exposure during infancy for this population.

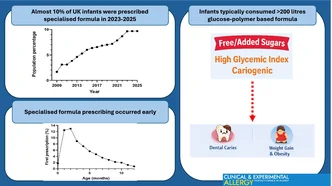

## 1 Introduction

Specialized formulae were developed in the early 20th century for managing formula‐fed infants with cow's milk allergy or malabsorption, but are also sometimes used for managing non‐specific symptoms in infants such as vomiting, loose or frequent stools or excessive crying [1]. Specialized formula differs from standard infant formula in both amino‐acid and carbohydrate composition. Amino‐acid sources are designed to be tolerated by children with cow's milk allergy—typically soya protein, extensively hydrolysed cow's milk protein or separate amino‐acids, and generally provide lower quality protein than standard cow's milk formula or breastmilk. Carbohydrate sources are typically glucose‐polymers, which are classified in the European Union as ‘free sugars’ and in the United States as ‘added sugars’ [2]. International dietary guidelines consistently recommend limiting or avoiding free/added sugars, especially during childhood [3]. Increasing use of specialized formula is therefore a concern, as has been reported in recent years in the United Kingdom, Australia, Norway and Ireland, due to the potential to promote dental decay, childhood obesity or other long‐term health conditions [4, 5, 6]. However, large‐scale population‐based estimates of the frequency and timing of specialized formula use are not yet available. We therefore used the United Kingdom's population‐based Clinical Practice Research Datalink (CPRD) Aurum database to estimate the frequency and timing of specialized formula use.

## 2 Methods

This study is a retrospective observational cohort study with quantitative descriptive analysis of secondary data using the CPRD Aurum database. CPRD Aurum is a de‐identified primary care database contributed to by UK general practices, primarily in England, and representing approximately 24% of the UK population [7]. During the study period, the database contained records for over 40 million patients meeting CPRD quality standards for research use. The CPRD population is broadly representative of the English population and includes patient demographic information and drug issue records containing primary care prescriptions [8].

### 2.1 Study Population

We included children who were born between January 2009 and December 2021, who registered with a general practice within their first 3 months of birth, with continuous registration for at least 2 years from their birth. In the UK, all infants are registered with a primary care centre (‘general practice’). Included children were classified as ‘acceptable’ by CPRD quality standards that is, year of birth not missing and record deemed of sufficient quality for use in research. The three‐month registration cut off was selected to ensure inclusion of the early months of infancy in the primary care record.

### 2.2 Study Exposure

Within the population of eligible children in CPRD Aurum born during the study period, we selected children prescribed specialized formula, classified as amino‐acid, extensively hydrolysed or soya formula [4, 5, 9]. In the UK, almost all specialized formula is obtained through primary care prescribing. Demographic characteristics, including age, gender and ethnicity were summarized. The CPRD Aurum drug issue files were used to determine prescriptions of specialized formula. A comprehensive list of specialized formula brands available in the UK during this study period was found using the CPRD product code browser. Included brands are shown in the Supporting Information (Table S1). We excluded products which are primarily used for managing malabsorption rather than allergy: Pregestimil, Nutrison/Fortison Soya, Cow & Gate Pepti‐Junior, Infantrini Peptisorb and Aptamil Pepti‐Junior; and we excluded products which are solid or semi‐solid foods rather than liquid or powdered formula products: Neocate Nutra and Neocate Spoon [4, 5].

### 2.3 Data Classification and Analysis

We aimed to evaluate, for each birth year, the proportion of children prescribed specialized formula before the age of 12 months, based on their age in months and their prescription record. To assess timing of initial prescriptions, the age in months at the first prescription was utilized. To assess volume and duration of exposure to specialized formula for individual patients, we also analysed the subset of children with repeat (i.e., more than one) specialized formula prescriptions. Duration of exposure could be estimated by calculating the number of days between the first and last prescription. Data distributions were evaluated using histograms, and parametric or non‐parametric descriptive statistics were used to describe data as appropriate. We used Spearman's rank correlation coefficient to evaluate potential changes over time. Prescription quantities were recorded in grams, millilitres or product pack sizes (sachets, tins, cans, boxes or packs). Pack‐based quantities were first converted to grams using product‐specific pack weights, taking the most commonly prescribed pack size where more than one was available. Grams were then converted to reconstituted volume using a single British National Formulary for Children reconstitution rate of 13.5 g of powder per 100 mL, applied to all specialized formula types [9]. The type and free sugars content of each specialized formula were collated using publicly available sources including manufacturer information and the British National Formulary for Children [9].

### 2.4 Estimation of Specialized Formula Frequency for Years 2022–2025

To estimate the frequency of specialized formula prescription in years 2022–2025, we first extracted the total quantity of specialized formula prescriptions for infants from 2009 to 2025 in England from NHS Business Services Authority (NHSBSA) using R script, as described previously [4]. To compare national specialized formula prescribing with birth rate, the total live birth number in England was extracted from the Office of National Statistics for the years 2009 to 2025 [10]. As CPRD Aurum is composed almost entirely of English practices, this analysis reflects prescribing in England. We used England‐level national prescribing and birth data accordingly. Annual prescribed litres of specialized formula were divided by the number of live births in the previous year to give a rate of specialized formula prescribing per liveborn infant. We then used the mean ratio between the national prescribing rate per liveborn infant and the proportion of infants within CPRD prescribed specialized formula, calculated for the prior 8 years (2014 to 2021), to estimate the proportion of infants within CPRD prescribed specialized formula in 2022 to 2025. National expected rate of community prescription of specialized formula was calculated using a method described previously [4].

### 2.5 Ethical Approval

CPRD has ethics approval from the National Research Ethics Service Committee (NHS HRA REC reference number: 05/MRE04/87). Imperial College London holds a multi‐study licence agreement for the use of CPRD data. Specific approval for this project was granted by the CPRD Research Data Governance (RDG) Process (protocol ID 24_004131; approval date 13/06/2024). Data management was provided by the Big Data and Analytical Unit (BDAU) at the Research Computing Services within Imperial. All data were securely stored within Imperial's BDAU Secure Environment and were accessible only to specified researchers (N.P., K.L., M.C.).

## 3 Results

### 3.1 Study Population and Products

There were 47,766,944 patients with acceptable data in the CPRD Aurum database, including 4,203,276 children born between 2009 and 2021, of whom 1,944,323 (46.3%) were included in the study (Figure 1). The specialized formula cohort comprised 111,478 children (5.7%), of whom 55.4% were male (compared with 51.2% of the total cohort), with a median age of 10 months. Demographic data including age, gender, and geographic location are shown in Table 1. Most characteristics were similar between the two groups, but the specialized formula cohort contained a higher proportion of boys (55.4% vs. 51.2%) and of children of White ethnicity (85.0% vs. 81.7% in the 2021 census comparator). There were 28 specialized formula products identified that were prescribed in CPRD (Table S1). This included 10 amino acid formula (AAF), 13 extensively hydrolysed formula (EHF), and 5 soya formula products. All products with available information about carbohydrate source used a glucose polymer (Table S1).

**FIGURE 1:**
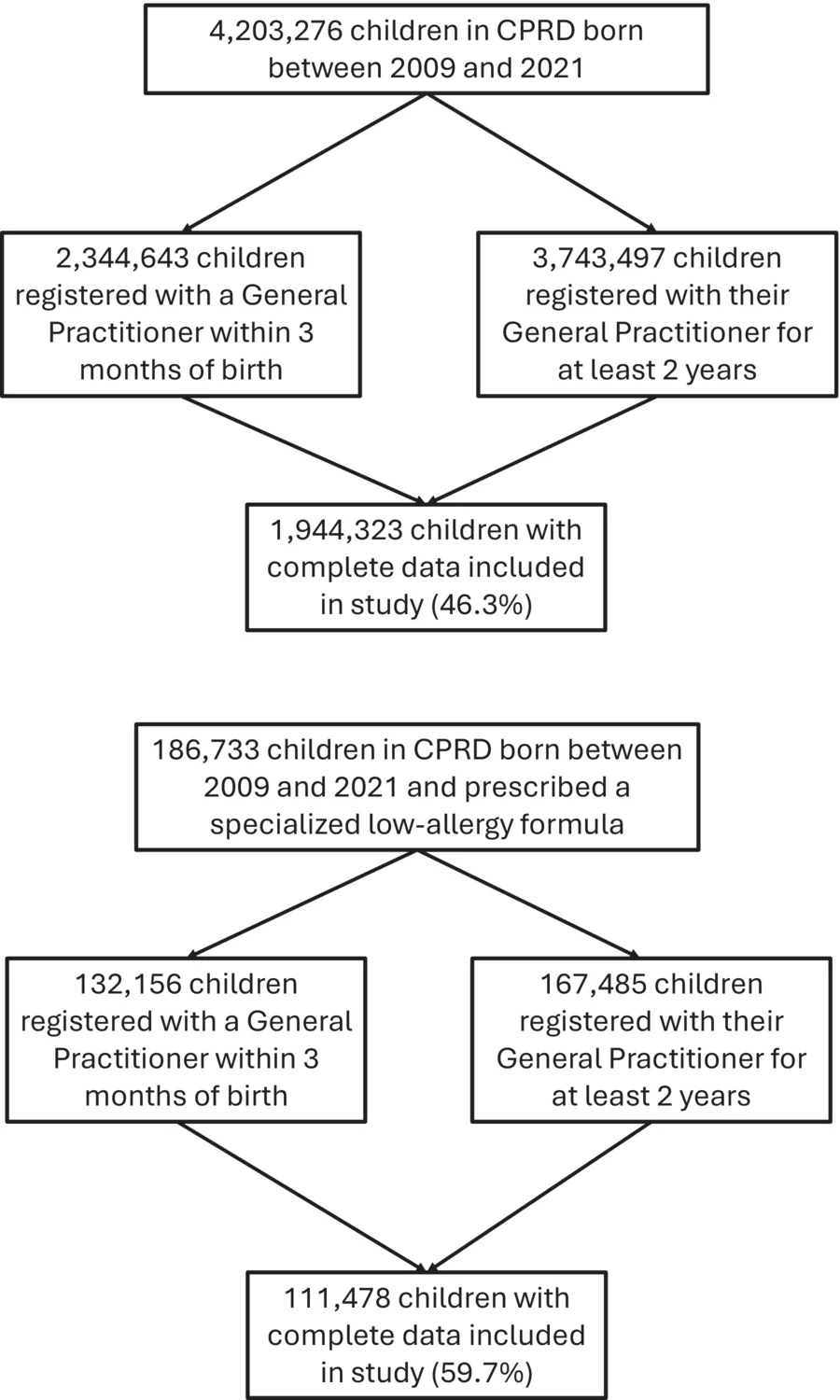
Study population. Both the denominator population and the numerator population were derived from the Clinical Practice Research Datalink Aurum (CPRD) database, a representative sample of primary care records across UK general practices, predominantly in England.

**TABLE 1:** Study population characteristics.

Characteristic	Specialized formula cohort (*n* = 111,478)	CPRD cohort (*n* = 1,944,323)
Median age (months)	10	11
Gender (%)		
Male	55.4	51.2
Female	44.6	48.8
Ethnicity (%)^a^		
White	85.0	81.7
South Asian	6.9	9.3
Black	2.4	4.0
Mixed	3.6	2.9
Other	2.2	2.1
Geographic location (%)		
North East	4.3	3.3
North West	19.8	18.6
Yorkshire and The Humber	3.1	3.0
East Midlands	2.2	2.0
West Midlands	14.6	16.2
East of England	4.5	3.8
London	16.1	21.1
South East	24.7	21.5
South West	9.3	9.6
Northern Ireland	1.4	0.6
Unknown	0	0.2

*Note:* Characteristics of CPRD cohort and specialized formula cohort including age, gender, ethnicity and geographic location data [7].

^a^ Ethnicity data of specialized formula population recorded *n* = 95,109 (85.3%). Ethnicity data could not be derived for the total CPRD population used in this study, due to technical limitations of the available data, so ethnicity data for England from the 2021 national census in 59.6 million people are shown for comparison.

### 3.2 Temporal Trend of Specialized Formula Prescribing

Between 2009 and 2025, the volume of specialized formula prescribed in England rose from 6.09 to 23.42 L per live birth, a 3.8‐fold increase (Figure 2). We estimated the expected volume, meaning the volume that would be prescribed if use were confined to infants with clinically confirmed cow's milk allergy [4]. This was 1.77 L per live birth in 2009 and 1.24 in 2025. Observed prescribing in 2025 was therefore 18.9 times higher than expected. From 2009 to 2021, the percentage of children under 1 year prescribed specialized formula increased from 1.65% (95% CI 1.58, 1.71) to 7.85% (95% CI 7.71, 7.99) (Figure 2). Percentage of children prescribed specialized formula for years 2022 to 2025 was estimated using the mean ratio of national prescribing rate to CPRD percentage of children prescribed for the prior 8 years (2014–2021: ratio mean 2.42, range 2.30 to 2.46) (Table S2). This yielded an estimate of 9.69% of children in CPRD prescribed a specialized formula in 2025 (Figure 2, Table S3).

**FIGURE 2:**
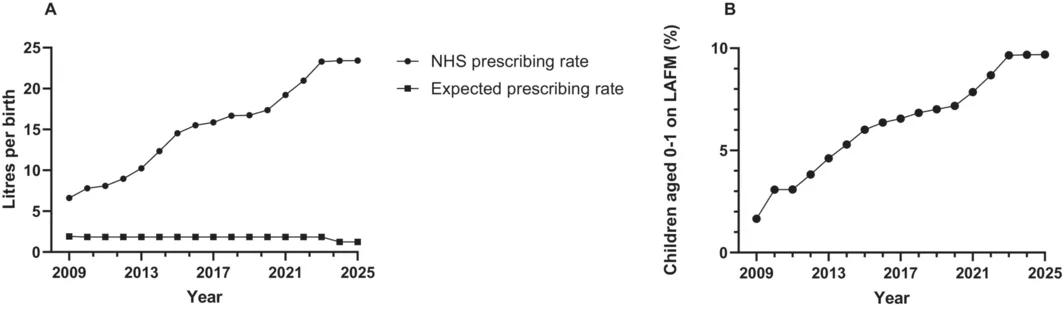
Increase in specialized formula prescribing over time. Increase in specialized formula prescribing in England from 2009–2025. Data show the volume of specialized formula prescribed in England for each live birth (A) and the estimated proportion of children aged 0–1 year prescribed specialized formula over time by year of birth (B). Volume was calculated using National Health Service (NHS) Prescribing data, live births for the previous calendar year from Office of National Statistics (ONS) birth statistics, proportion of children from CPRD from 2009–2021. From 2022–2025, we estimated the proportion of children using the NHS Prescribing volumes, based on the median volume per child prescribed specialized formula in CPRD from 2009–2021.

### 3.3 Timing of Specialized Formula Prescribing

Timing of first specialized formula prescription is shown in Figure 3. The highest rate of initiation occurred in the first 2 months of life, where 25% of first prescriptions were initiated. Initiation rates gradually decreased to less than 1% of affected infants in the 12th month of life (Figure 3, Table S4). The age with highest prevalence of specialized formula prescribing was from 6–10 months of age. At these ages, in any 1 month over one‐third of those prescribed a specialized formula had an active prescription (Table S5).

**FIGURE 3:**
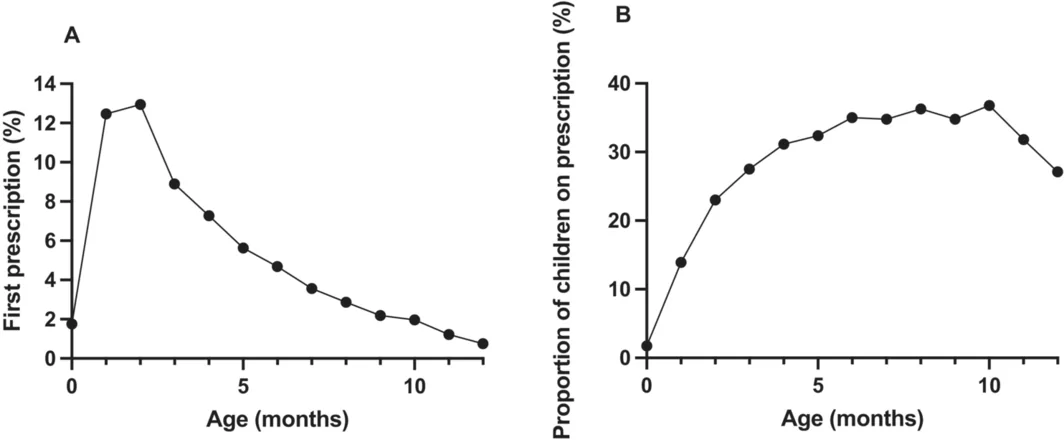
Age of children at prescription of specialized formula. Age distribution of children prescribed specialized formula. (A) Percentage of children prescribed their first prescription of specialized formula, by age in months at initiation. (B) Proportion of all CPRD children with a prescription for specialized formula, by age in months.

### 3.4 Temporal Change in Composition of Specialized Formula Prescribed for CPRD Population

During the period 2009 to 2021, the proportion of infant first prescriptions that was an extensively hydrolysed formula increased from 50.2% to 84.0%, while the proportion first prescribed soya formula decreased from 34.1% to 0.8% and first prescription of amino‐acid formula remained stable at about 15.0% (Table S3; Figure S1). During the study period, the carbohydrate and protein composition of specialized formula prescribed to the CPRD population changed. There was a significant increase in prescribed volume of maltodextrin‐based (Figure 4A) and amino‐acid‐based (Figure 4B) specialized formulas over time.

**FIGURE 4:**
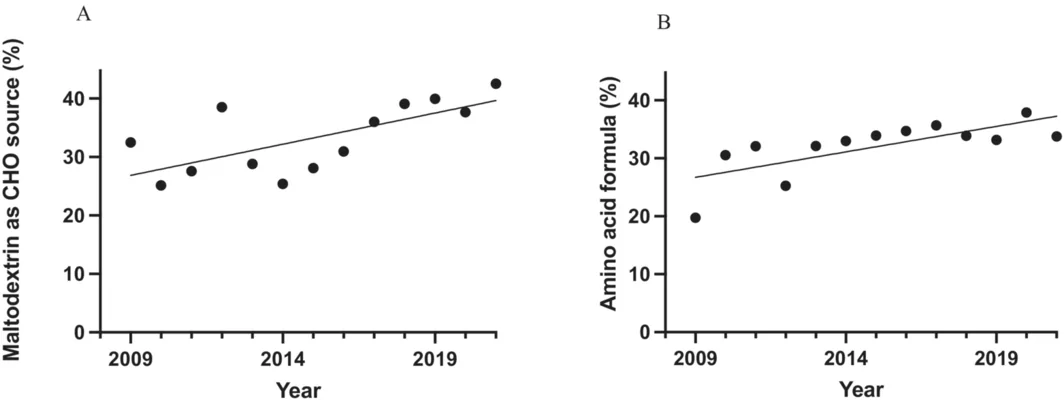
Change in composition of specialized formula over time. Data show the proportion of specialized formula with maltodextrin as the main carbohydrate source, as total volume prescribed in the CPRD population (A) and proportion of amino‐acid formula prescribed within the total specialized formula volume prescribed in the CPRD population (B). The increase over time was statistically significant for maltodextrin (Spearman rank correlation coefficient *r* = 0.69 95% CI 0.20, 0.90, *p* = 0.01) / (slope = 1.07, 95% CI = 0.33, 1.81, *p* < 0.01) and for amino‐acid formula (Spearman rank correlation coefficient *r* = 0.79 95% CI 0.41, 0.94, *p* < 0.01) / (slope = 0.88, 95% CI 0.33, 1.43, *p* < 0.01). Mean proportions of specialized formula with maltodextrin as the main carbohydrate source were significantly higher in 2015–2021 compared with 2009–2014 (Welch's *t* = −2.34, *p* = 0.04).

### 3.5 Total Volume and Duration of Specialized Formula Prescribing for Children in CPRD

We estimated the total volume and duration of specialized formula exposure for children prescribed specialized formula on more than one occasion. This population was composed of 85,370 children, 76.6% of the CPRD population prescribed a specialized formula on at least one occasion. Duration of exposure was longest for amino‐acid formula (median 257 days) compared with extensively hydrolysed formula (median 213 days) and shortest for soya formula (median 152 days). Mean total volume consumed during this period per child was 242 L (amino‐acid), 203 L (extensively hydrolysed) and 137 L (soya formula; Table 2).

**TABLE 2:** Exposure to specialized formula: Prescribed volume, age at initiation and duration.

Type of formula	Children with > 1 prescription	Total volume prescribed (L)	Mean volume per child (L)	Median age at initiation (months, IQR)	Median duration of exposure (days, IQR)
Amino acid	26,498	6,411,232	242	3 (2, 6)	257 (149, 341)
Extensively hydrolysed	58,034	11,788,496	203	3 (2, 5)	213 (131, 322)
Soya	5279	724,890	137	5 (3, 8)	152 (50, 268)

*Note:* Data are shown for children prescribed specialized formula of the same category on more than one occasion.

## 4 Discussion

In this population‐based study, we found that over a 12‐year period, there was a 5‐fold increase in the proportion of children prescribed a specialized formula. Our estimates suggest that up to 10% of infants in England may have been prescribed a specialized formula in 2025. The data show that the first 2 months of life are the highest‐rate period for first prescription of specialized formula, accounting for about one quarter of initiations. Prescriptions continue for 4–8 months, depending on the type of formula used. Affected infants may consume over 200 L of glucose‐polymer based formula during this time. These findings are likely to reflect a global pattern of increasing exposure of young infants to glucose‐polymer based specialized formula products [5]. The pattern is concerning, given the long‐term health risks associated with free/added sugars consumption in early life [11, 12].

Specialized formula products are developed and regulated as suitable for the management of cow's milk allergy. Cow's milk allergy affects about 1% of infants, but we estimate 10% of infants were prescribed a specialized formula in England in 2025 [13]. Thus, most specialized formula use documented in this study was for infants without cow's milk allergy. This is outside of the approved indication for such products, but is consistent with our previous finding in a less representative population that most specialized formula use is for children who do not have cow's milk allergy, consistent with overdiagnosis of the condition [1].

Initiation of specialized formula prescribing in this population‐based dataset most commonly occurred in the first 2 months, whereas in Europrevall, immunoglobulin E (IgE)‐mediated and non‐IgE mediated cow's milk allergy was most commonly confirmed after age 6 months [13]. This suggests that most specialized formula prescribing in this study is for non‐specific symptoms of infancy, which peak in prevalence in the first 2 months [14]. Our previous analysis of primary care records for a smaller cohort identified gastrointestinal symptoms such as vomiting, crying and loose stools as the most common triggers for milk allergy overdiagnosis and specialized formula prescription [1]. Clinical practice guidelines have been criticized for promoting the concept that common, early, non‐specific symptoms which occur in most infants are signs of cow's milk allergy [15].

The health and developmental effects of specialized formula consumption during infancy are not well established. Glucose‐polymers comprise some or all of the carbohydrate source for all specialized formula products, together with maltodextrin, sucrose, fructose and/or lactose. Glucose‐polymers differ from the lactose present in human milk, cow's milk or standard infant formula, and are classified by most public health authorities as ‘added’ or ‘free’ sugars [2, 3, 16]. These sugars typically have a high glycemic index which can lead to insulinemia and adipose deposition and are cariogenic [17, 18]. Child obesity and dental decay are important public health issues so the increasing exposure of infants and young children to specialized formula is potentially of public health importance [18]. Evidence from the US suggests that glucose polymer based infant formula may increase risk for early childhood obesity [19]. Natural experiment studies evaluating the impact of sugar rationing following World War II have identified links between exposure to sugars in the first 2 years and longer‐term risk for a range of health conditions including obesity, diabetes mellitus and cardiovascular disease during adult life [11, 12]. Substitution of lactose with glucose‐polymers also partly or completely removes galactose from the infant diet. It is not known whether galactose deprivation during early life has any specific consequences for human brain development [20, 21].

We identified a shift away from soya formula and towards maltodextrin‐dominant and amino‐acid‐based formula products. There was a significant increase in maltodextrin‐based formula volume after 2015, when the World Health Organization classification of free sugars was published which, unlike the United States definition of added sugars, excluded maltodextrin from the definition [3, 22]. The changes in product types prescribed over time may relate to clinical practice guidelines which advise against soya formula and regulatory approaches to classifying maltodextrins versus glucose syrups [16].

This is the first population‐based description of the frequency and timing of specialized formula prescribing and confirms previous suggestions of early initiation and increasing prescription of these products. CPRD Aurum is broadly representative of the English population, so the estimates are likely to be generalizable to England. Prescribing patterns, and their socioeconomic and geographical distribution, may differ in the devolved nations, which have different populations, levels of deprivation and health policies [23]. Comparison across the four UK nations, and analysis of variation by deprivation and between rural and urban areas, were beyond the descriptive scope of this study and are important directions for future work using data sources with fuller coverage of Scotland, Wales and Northern Ireland. Our previous work identified increasing specialized formula prescribing in other high‐income settings including Norway, suggesting these patterns are likely to be relevant to other European countries. However, the precise frequency and rate of change may not be generalizable, especially to lower income settings [5]. As with other real‐world datasets, this study is also subject to the limitations of a retrospective design using routinely collected primary care data. It relies on information entered into electronic health records, cannot establish the clinical indication for individual prescriptions, and is subject to the completeness and accuracy of record entry. Children who did not register with a general practice within 3 months of birth or who did not have continuous registration for 2 years, were excluded. These children may differ systematically from those included, for example in patterns of migration or health‐service contact, which may affect the generalizability of our estimates. We classified products by their primary licensed indication, following our previous work [5]. The prescription record does not capture the clinical indication for an individual prescription, so some misclassification of product use is possible. The specialized formula cohort showed a male predominance and a higher proportion of children of White ethnicity than the comparison populations, though these observations should be interpreted cautiously given that the study is descriptive and was not designed as an analytic comparison of the prescribed and non‐prescribed groups, and that the ethnicity comparator is the national census rather than the CPRD cohort.

## 5 Conclusions

In conclusion, we have identified a striking increase in specialized formula prescribing in England, with up to 10% of infants in England receiving a prescription in 2025. The change is likely to represent increasing use of specialized formula for non‐specific symptoms during early infancy. This may have an important public health impact due to the associated high levels of free sugars exposure during early life.

## Author Contributions

Nikita Punjabi, Karen Li, Mark Cunningham and Robert J. Boyle had access to the data in the study and take responsibility for the integrity of the data and the accuracy of the data analysis. Concept and design: Robert J. Boyle, Nikita Punjabi, Professor Hargreaves, Professor Quint. Acquisition, analysis or interpretation of data: All authors. Drafting of the manuscript: Nikita Punjabi, Karen Li, Robert J. Boyle. Critical revision of the manuscript for important intellectual content: All authors. Statistical analysis: Karen Li, Robert J. Boyle, Mark Cunningham. Administrative, technical or material support: All authors. Study supervision: Robert J. Boyle.

## Funding

This research study was co‐funded by the NIHR Imperial Biomedical Research Centre (BRC) and by a NIHR Academic Clinical Fellowship (ACF) awarded to Nikita Punjabi. The views expressed are those of the author(s) and not necessarily those of the NIHR or the Department of Health and Social Care.

## Conflicts of Interest

Dr. Boyle reports payments for editorial work from Wiley, Cochrane and the British Society for Allergy and Clinical Immunology, for expert witness work in legal cases related to food anaphylaxis and infant formula health claims, for consultancy to the World Health Organization and the Norwegian Directorate for Health. The remaining authors declare no conflicts of interest.

## Supporting information


**Figure S1:** Type of specialized formula prescribed for children in their first year.
**Table S1:** Specialized formula products used in the population‐based CPRD dataset between 2009 and 2021.
**Table S2:** Estimation of specialized formula prescribing frequency in 2022–2024.
**Table S3:** Proportion of children aged 0–1 prescribed specialized formula in CPRD dataset.
**Table S4:** Proportion of children initiated on specialized formula during the first year.
**Table S5:** Proportion of children on specialized formula during the first year.

## Data Availability

This study is based in part on data from the Clinical Practice Research Datalink (CPRD) obtained under licence from the UK Medicines and Healthcare products Regulatory Agency. The data is provided by patients and collected by the UK National Health Service (NHS) as part of their care and support. The interpretation and conclusions contained in this study are those of the authors alone. CPRD has NHS Health Research Authority (HRA) Research Ethics Committee (REC) approval to allow the collection and release of anonymized primary care data for observational research [NHS HRA REC reference number: 05/MRE04/87], this approval was granted on the 11/02/2025. Each year CPRD obtains Section 251 regulatory support through the HRA Confidentiality Advisory Group (CAG), to enable patient identifiers, without accompanying clinical data, to flow from CPRD contributing GP practices in England to NHS Digital, for the purposes of data linkage [CAG reference number: 21/CAG/0008]. The protocol for this research was approved by CPRD's Research Data Governance (RDG) Process (protocol number: 24_004131) and the approved protocol is available here https://www.cprd.com/approved‐studies/prevalence‐and‐factors‐associated‐low‐allergy‐formula‐milk‐prescription‐england. Select general practices consent to this process at a practice level with individual patients having the right to opt‐out. Data are available on request from the CPRD. Their provision requires the purchase of a licence, and this licence does not permit the authors to make them publicly available to all. This work used data from the version collected in June 2024 and has clearly specified the data selected within each Methods section. To allow identical data to be obtained by others, via the purchase of a licence, the code lists can be provided upon request. Licences are available from the CPRD (http://www.cprd.com): The Clinical Practice Research Datalink Group, The Medicines and Healthcare products Regulatory Agency, 10 South Colonnade, Canary Wharf, London E14 4PU.
