# Supplementary material for: Frequency and Timing of Specialized Formula Prescriptions for Children Under 2 Years
Source: Clin Exp Allergy. 2026 Oct 7;56(11):1411–8. doi: 10.1111/cea.70447 (PMC13727345; doi:10.1111/cea.70447)
Supplement: Supplementary file 1 [file CEA-56-1411-s001.docx]

**Frequency and timing of specialized formula prescriptions for children under 2 years**

Nikita Punjabi MD, PhD^1^, Karen Li MD^1^, Mark Cunningham MSc^1^, Kimberley Foley PhD^1^, Frank Moriarty PhD^2^, Sonia Saxena MD, PhD^1^, Jennifer K Quint MD, PhD^1^, Dougal Hargreaves MD, PhD^1^, Robert J Boyle MD, PhD^1^

^1^School of Public Health, Imperial College London, W12 0BZ UK, ^2^School of Pharmacy and Biomolecular Sciences, RCSI University of Medicine and Health Sciences, Dublin, Ireland.

**Address for Correspondence:**
Robert J. Boyle, MB ChB, PhD
Imperial College School of Public Health

90 Wood Lane

London W12 0BZ Tel: +44 207 594 3990 Email: [r.boyle@imperial.ac.uk](mailto:r.boyle@imperial.ac.uk)

ORCID 0000-0002-4913-7580

**Table of Contents**

[**Figure S1. Type of specialized formula prescribed for children in their first year	3**](#_Toc221637894)

[**Table S1. Specialized formula products used in the population-based CPRD dataset between 2009 and 2021	4**](#_Toc221637895)

[**Table S2. Estimation of specialized formula prescribing frequency in 2022-2024	5**](#_Toc221637896)

[**Table S3. Proportion of children aged 0-1 prescribed specialized formula in CPRD dataset	6**](#_Toc221637897)

[**Table S4. Proportion of children initiated on specialized formula during the first year	7**](#_Toc221637898)

[**Table S5. Proportion of children on specialised formula during the first year	8**](#_Toc221637899)

## Figure S1. Type of specialized formula prescribed for children in their first year

Data show total volume of each category of specialized formula prescribed in the CPRD cohort (**A**) and percentage of children aged 0-1 of the CPRD cohort on specialized formula and the type of formula they were first prescribed (**B**) from 2009-2021. EHF: extensively hydrolysed formula. AAF: amino acid formula.

## Table S1. Specialized formula products used in the population-based CPRD dataset between 2009 and 2021

| **Type of formula** | **Study Years Prescribed** | **Carbohydrate source(s)** | **Total non-lactose carbohydrate content (g per 100ml reconstituted)** |
| --- | --- | --- | --- |
| **Amino acid formula** | | | |
| Neocate | 2009-2019 | Maltodextrin | 7.3 |
| Neocate active | 2009-2018 | Not specified - ‘carbohydrate’ | Not available |
| Neocate advance | 2009-2022 | Not specified - ‘carbohydrate’ | 14.6 |
| Neocate LCP | 2009-2022 | Dried glucose syrup | 7.1 |
| Neocate Junior (for 1+) & vanilla | 2017-2022 | Dried glucose syrup, maltodextrin | 10.7-11.2 (flavoured) |
| Neocate Syneo | 2018-2022 | Dried glucose syrup, oligosaccharides | 7.2 |
| SMA Alfamino | 2014-2022 | Glucose syrup, starch | 7.5 |
| Nutramigen PurAmino | 2009-2022 | Glucose syrup, modified tapioca starch | 6.9 |
| Nutaminigen AA Lipil | 2009-2015 | Glucose syrup, modified tapioca starch | Not available |
| Elecare powder | 2020-2022 | Corn syrup solids | 7.2 |
| **Extensively hydrolysed formula** | | | |
| Nutramigen | 2009-2012 | Not available | Not available |
| Nutramigen Lipil 1 | 2009-2019 | Glucose syrup, modified corn starch | Not available |
| Nutramigen Lipil 2 | 2009-2020 | Glucose syrup, modified corn starch | Not available |
| Nutramigen 1 with LGG powder | 2011-2022 | Glucose syrup, modified corn starch | 7.4 |
| Nutramigen 2 with LGG powder | 2015-2022 | Glucose syrup, fructose, modified corn starch | 7.7 |
| Nutramigen 3 (with LGG powder (1+)) | 2018-2022 | Glucose syrup, fructose, modified corn starch | 7.7 |
| SMA Althera | 2013-2022 | Maltodextrin, lactose | 3.6 |
| Similac Alimentum | 2013-2020 | Maltodextrin, sucrose, modified corn starch | 6.6 |
| Aptamil Pepti 1 | 2010-2022 | Maltodextrin, lactose, oligosaccharides | 4.2 |
| Aptamil Pepti 2 | 2012-2022 | Maltodextrin, lactose, oligosaccharides | 4.8 |
| Aptamil Pepti Syneo | 2021-2022 | Maltodextrin, lactose, oligosaccharides | 4.2 |
| Cow and Gate Pepti | 2009-2010 | Maltodextrin, lactose, oligosaccharides | 4.2 |
| Milupa Prejomin | 2009 | Not available | Not available |
| **Soya formula** | | | |
| Wysoy | 2009-2022 | Maltodextrin | 6.8 |
| SMA Soya | 2012-2022 | Maltodextrin | 6.8 |
| Farley’s Soya | 2009-2011 | Not available | Not available |
| Infasoy | 2009-2015 | Glucose Syrup | 7 |
| Soya (unspecified) | 2009-2014 | Not available | Not available |

Products documented in the CPRD dataset as having been prescribed to infants between 2009 and 2021, categorized according to their protein and carbohydrate sources.

## Table S2. Estimation of specialized formula prescribing frequency in 2022-2025

| **Year** | **Total volume prescribed (L, England)** | **Live births in previous year (England)** | **Volume prescribed per birth (L)** | **Proportion of infants prescribed in CPRD dataset** | **Ratio of volume per birth nationally to proportion in CPRD** |
| --- | --- | --- | --- | --- | --- |
| 2014 | 8272285 | 664,517 | 12.45 | 5.29 | 2.35 |
| 2015 | 9687962 | 661,496 | 14.65 | 6.01 | 2.44 |
| 2016 | 10404458 | 664,399 | 15.66 | 6.36 | 2.46 |
| 2017 | 10631871 | 663,157 | 16.03 | 6.56 | 2.45 |
| 2018 | 10904313 | 646,794 | 16.86 | 6.84 | 2.46 |
| 2019 | 10571731 | 657,076 | 16.09 | 7.01 | 2.30 |
| 2020 | 10605773 | 610,505 | 17.37 | 7.18 | 2.42 |
| 2021 | 11251268 | 585,195 | 19.23 | 7.85 | 2.45 |
| 2022 | 12494325 | 595,948 | 20.97 | - | - |
| 2023 | 13450891 | 577,046 | 23.31 | - | - |
| 2024 | 13185333 | 563,561 | 23.40 | - | - |
| 2025 | 13297299 | 567,708 | 23.42 |  |  |

Method used for estimating frequency of specialized formula prescribing in 2022-2025. The proportion of infants in the CPRD dataset prescribed a specialized formula each year was compared with the volume of specialized formula prescribed per child born in the previous year nationally. This ratio was relatively stable from 2014 at mean 2.42 (range 2.30 to 2.46). So the ratio 2.42 was used to estimate proportion of infants in the CPRD dataset prescribed a specialized formula in 2022 to 2025, based on national prescribing data available for those years.

## Table S3. Proportion of children aged 0-1 prescribed specialized formula in CPRD dataset

| **Year** | **Children prescribed specialized formula** | **All children born in that year** | **Percentage of children prescribed specialized formula (%)** | **Percentage first prescribed extensively hydrolysed formula (EHF) (%)** | **Percentage first prescribed amino acid formula (AAF) (%)** | **Percentage first prescribed soya formula (%)** |
| --- | --- | --- | --- | --- | --- | --- |
| 2009 | 2385 | 144968 | 1.65 | 50.19 | 15.77 | 34.05 |
| 2010 | 4640 | 150795 | 3.08 | 49.57 | 17.05 | 33.38 |
| 2011 | 4688 | 151896 | 3.09 | 50.60 | 21.29 | 28.11 |
| 2012 | 5941 | 155421 | 3.82 | 59.92 | 19.91 | 20.16 |
| 2013 | 6921 | 150229 | 4.61 | 68.89 | 17.27 | 13.84 |
| 2014 | 8045 | 152156 | 5.29 | 72.57 | 17.63 | 9.81 |
| 2015 | 9195 | 153087 | 6.01 | 77.30 | 16.32 | 6.37 |
| 2016 | 9842 | 154754 | 6.36 | 79.64 | 16.56 | 3.80 |
| 2017 | 10002 | 152591 | 6.55 | 83.29 | 14.52 | 2.19 |
| 2018 | 10347 | 151193 | 6.84 | 85.30 | 13.21 | 1.49 |
| 2019 | 10356 | 147732 | 7.01 | 86.73 | 12.17 | 1.10 |
| 2020 | 9815 | 136619 | 7.18 | 84.55 | 14.34 | 1.11 |
| 2021 | 11220 | 142882 | 7.85 | 83.98 | 15.28 | 0.75 |
| 2022 | - | - | 8.68 | - | - | - |
| 2023 | - | - | 9.65 | - | - | - |
| 2024 | - | - | 9.68 | - | - | - |
| 2025 | - | - | 9.69 | - | - | - |

CPRD data were extracted for 2009-2021. Estimates for the years 2022 to 2025 were calculated as shown in Table S2.

## Table S4. Proportion of children initiated on specialized formula during the first year

| Age (months) | Number of first-time specialized formula prescriptions | Number of children in study | Proportion of first-time specialized formula prescriptions (%) |
| --- | --- | --- | --- |
| 0 | 2814 | 159813 | 1.76 |
| 1 | 19991 | 160413 | 12.46 |
| 2 | 22022 | 170171 | 12.94 |
| 3 | 14959 | 168017 | 8.9 |
| 4 | 12067 | 165980 | 7.27 |
| 5 | 9560 | 169675 | 5.63 |
| 6 | 7577 | 161831 | 4.68 |
| 7 | 5831 | 163891 | 3.56 |
| 8 | 4463 | 155937 | 2.86 |
| 9 | 3512 | 160503 | 2.19 |
| 10 | 2885 | 147499 | 1.96 |
| 11 | 1956 | 160593 | 1.22 |
| 12 | 1204 | 159812 | 0.75 |

Data show the proportion of children prescribed specialized formula for the first time, for each month for the first year of life, in the CPRD dataset.

## Table S5. Proportion of children on specialized formula during the first year

| Age (months) | Number of children prescribed specialized formula at any time | Number of children in study | Proportion of children on specialized formula (%) |
| --- | --- | --- | --- |
| 0 | 2814 | 159813 | 1.76 |
| 1 | 22312 | 160413 | 13.91 |
| 2 | 39184 | 170171 | 23.03 |
| 3 | 46240 | 168017 | 27.52 |
| 4 | 51721 | 165980 | 31.16 |
| 5 | 54956 | 169675 | 32.39 |
| 6 | 56695 | 161831 | 35.03 |
| 7 | 56996 | 163891 | 34.78 |
| 8 | 56574 | 155937 | 36.28 |
| 9 | 55827 | 160503 | 34.78 |
| 10 | 54245 | 147499 | 36.78 |
| 11 | 51093 | 160593 | 31.82 |
| 12 | 43344 | 159812 | 27.12 |

Data show the proportion of children prescribed specialized formula for each month in the first year of life, in the CPRD dataset.
